# Protein-mediated stabilization of amphotericin B increases its efficacy against diverse fungal pathogens

**DOI:** 10.1128/spectrum.00686-25

**Published:** 2025-04-15

**Authors:** Kenya E. Fernandes, Caitlin L. Johnston, Brayden C. Williams, Dee A. Carter, Margaret Sunde

**Affiliations:** 1 School of Life and Environmental Sciences, University of Sydney 4334, Sydney, New South Wales, Australia; 2 Sydney Institute for Infectious Diseases, University of Sydney 4334, Sydney, New South Wales, Australia; 3 School of Medical Sciences and Sydney Nano, University of Sydney 4334, Sydney, New South Wales, Australia; University of Texas Medical Branch at Galveston, Galveston, Texas, USA

**Keywords:** amphotericin B, hydrophobins, antifungals, combination therapy

## Abstract

Amphotericin B (AMB), a potent and broad-spectrum antifungal agent, faces solubility and toxicity challenges in clinical use. In this study, we explored the ability of DewY and EAS_Δ15,_ class I fungal hydrophobin proteins with unique amphipathic properties and self-assembly capabilities, to stabilize AMB in solution. UV-visible spectroscopy confirmed the ability of hydrophobin proteins to stabilize the monomeric state of AMB in aqueous solution for up to 48 h. Further assays revealed that this effect was not exclusive to hydrophobins, however, as non-hydrophobin proteins provided similar stabilizing effects. AMB-protein combinations exhibited enhanced efficacy against diverse clinically relevant fungal pathogens, with 4- to 32-fold reductions in the effective *in vitro* dosage compared to AMB alone. Microscopic analyses found fungal cells treated with AMB alone and in combination with proteins had identical morphological changes, suggesting that protein interactions do not alter the mode of action of AMB. Instead, our results indicate that the monomeric state of AMB is stabilized in aqueous solution by non-specific interactions with hydrophobic areas on proteins. We suggest that this protein-mediated enhancement of solubility could reduce the required dose of AMB, providing a basis for optimizing AMB-based antifungal therapies.

**IMPORTANCE:**

Fungal infections are a growing global health concern, yet effective antifungal treatments remain limited by toxicity and poor solubility. AMB, a potent broad-spectrum antifungal, is highly effective but suffers from severe side effects and formulation challenges. Our study demonstrates that proteins, including fungal hydrophobins, can stabilize AMB in its monomeric form, significantly enhancing its solubility and efficacy against a range of fungal pathogens. These findings suggest that protein-mediated stabilization could enhance the effectiveness of AMB by reducing the required dosage and potentially lowering its toxic side effects. This approach offers a promising strategy for optimizing AMB therapies and improving treatment options, especially in resource-limited settings where fungal infections impose a significant health burden.

## INTRODUCTION

Amphotericin B (AMB) is a potent polyene antifungal drug used to treat life-threatening fungal infections. Its broad-spectrum efficacy arises from its ability to bind ergosterol in fungal membranes, disrupting membrane integrity (1). However, the clinical use of unmodified AMB is hampered by severe nephrotoxicity and poor aqueous solubility (2). Formulation with sodium deoxycholate improves solubility but is still associated with dose-limiting toxicities. Lipid- and liposomal-based formulations have been developed to mitigate toxicity and improve delivery. However, while these formulations have improved therapeutic outcomes, their very high production cost restricts their accessibility (3, 4). Recently, research into AMB analogs has identified new renal-sparing derivatives, although challenges related to formulation and production costs are likely to persist (5).

The growing global burden of fungal infections, exacerbated by the increasing immunocompromised population and the potential emergence of novel pathogens linked to global change, underscores the urgent need for accessible and effective antifungal therapies (6). This need is particularly acute in resource-limited regions, where fungal infections impose significant morbidity and mortality. Addressing this requires new and affordable broad-spectrum antifungal solutions. One promising approach is synergistic therapy, which combines two or more agents to enhance efficacy while lowering drug doses, thereby reducing toxicity and off-target effects. Recent studies have shown the potential of AMB combined with various agents, including other antifungal drugs (7), repurposed medications (8), proteins (9), and peptides (10). These findings provide promising leads for therapeutic development and can deepen our understanding of the molecular mechanisms underlying the biological activity of AMB (4).

A key challenge in optimizing AMB therapy lies in its propensity to aggregate and present as both monomeric and multimeric forms, each with distinct biological properties (11). Studies have shown that AMB aggregates are toxic to both erythrocytes and fungal cells, while monomers are selectively toxic to fungal cells (12, 13). Modifying AMB to increase its critical aggregation concentration has shown potential for reducing hemolytic activity and increasing selectivity for fungal cells (14). Therefore, exploring combinatory agents that could reduce aggregation and stabilize the monomeric form of AMB in solution may offer a promising strategy to improve its therapeutic profile.

Fungal hydrophobins are a family of small, amphipathic proteins produced exclusively by fungi. Hydrophobins self-assemble into monolayers that modify interfaces, contributing to fungal biology by making surfaces hydrophobic and non-immunostimulatory (15–17). These proteins exhibit a large exposed hydrophobic surface area, which underpins their surface activity and ability to interact with hydrophobic molecules (18–20). Class I hydrophobins form amyloid-structured fibrils known as rodlets, which pack into amphipathic monolayers (17, 18). Notably, hydrophobins are non-immunostimulatory in their isolated, purified form, showing no activation of dendritic cells, alveolar macrophages, or helper T-cell immune responses (16) and are widely consumed in edible mushrooms without adverse effects (21). These attributes have led to significant interest in their potential medical and biotechnological applications. Hydrophobins have been shown to stabilize hydrophobic compounds in solution and to improve the stability of drug-loaded liposomes (15, 22, 23). They have also been shown to be non-cytotoxic across different cell types and upon intravenous delivery (24, 25). They could therefore offer a novel approach to overcoming the challenges associated with hydrophobic drugs like AMB.

We have previously reported that the class I hydrophobin DewY (from *Aspergillus nidulans*) stabilizes AMB in aqueous solution as a rodlet-coated nanoparticle form (26). Here, we sought to evaluate how DewY and another class I hydrophobin, EAS_Δ15_ (from *Neurospora crassa* [19]), might affect the antifungal activity of AMB. We found that these hydrophobins effectively stabilized AMB in solution and increased its potency. However, our investigation of the physicochemical basis for this interaction revealed that other proteins could stabilize AMB, suggesting that the effect may stem from transient, non-specific interactions with hydrophobic areas on protein surfaces. Our results suggest that protein-mediated stabilization of AMB could enhance its therapeutic profile by improving solubility and reducing the dose required for effective antifungal activity.

## MATERIALS AND METHODS

### Fungal strains and culture conditions

Three yeast species (*Saccharomyces cerevisiae* S288c, *Cryptococcus neoformans* H99, and *Candida albicans* SC5314) and two mold species (*Aspergillus fumigatus* ATCC204305 and *Mucor circinelloides* 80–14-240-5007) were used for antifungal testing. Strains were maintained as glycerol stocks at −80°C, grown on potato dextrose agar (Oxoid), and incubated at 30°C for 24–48 h before use.

### Protein production and purification

Hydrophobin proteins DewY and EAS_Δ15_ were produced recombinantly as His_6_-ubiquitin-tagged fusion proteins, with EAS_Δ15_ purified as previously described (26). DewY was purified under native conditions via Ni-NTA affinity chromatography, followed by cleavage from the fusion partner, and finally purification by reverse phase high-performance liquid chromatography. Purified DewY and EAS_Δ15_ were lyophilized and stored at –20°C until use. The cleaved His_6_-ubiquitin (Ub) produced as a by-product of DewY purification was stored under the same conditions and used subsequently for AMB + Ub formulations. Bovine α-lactalbumin (α-lac, L6385), bovine serum albumin (BSA), and thioflavin T (ThT) were purchased from Sigma-Aldrich (St. Louise, MO, USA).

### Formulation of drug and protein samples

AMB, fluconazole (FLC, Sapphire Bioscience) and nystatin (NYS, Sigma-Aldrich) were solubilized in DMSO, and the deoxycholate formulation of AMB (AMB-D, Sigma-Aldrich) and caspofungin (CAS, Sigma-Aldrich) were solubilized in MilliQ water at 100 µg/mL. Lyophilized DewY, EAS_Δ15_, Ub, BSA, and α-lac were dissolved in MilliQ water at a concentration of 50 or 100 µg/mL. For drug-protein samples, the drug master stock was added to solubilized protein solution to achieve a final drug concentration of 1 µg/mL (1% dimethyl sulfoxide [vol/vol]) in glass vials. All drug-protein samples were mixed gently, except for the AMB shaken/rodlet samples, which were vigorously mixed for 1 h to promote the assembly of DewY rodlets at air-water interfaces.

### AMB stability monitoring by UV-visible spectroscopy

AMB stability in the presence or absence of proteins was assessed using UV-visible (UV-vis) spectroscopy. Following preparation, AMB formulations were transferred to a cuvette, and absorbance spectra were immediately measured (300–450 nm) using a Beckman DU 800 spectrophotometer (Beckman Coulter). Samples requiring 1 h incubation at room temperature were placed back into vials, and the spectra were collected again after the appropriate time.

### ThT fluorescence kinetic assays

Lyophilized DewY was dissolved in MilliQ water at 50 µg/mL and added to a 96-well plate with 40 µM ThT in the presence or absence of AMB, CAS, or FLC at 1 µg/mL. Rodlet assembly was initiated by vigorously shaking the plate at 700 rpm in a POLARstar Omega plate reader (BMG Labtech). Fluorescence intensity was measured at room temperature with constant shaking over 100 min using excitation/emission filters of 440/480 nm, respectively.

### Transmission electron microscopy

Samples containing DewY in the presence and absence of AMB were retrieved from the ThT assay, and 10 µL of each was floated on standard Formvar on carbon film-coated transmission electron microscopy grids (ProScitech) for 4 min. Excess liquid was removed by touching the grid to filter paper. Grids were washed with water and stained by floating on 2% uranyl acetate solution for 4 min, then air-dried. Grids were examined and imaged using an FEI Tecnai T12 microscope operating at 120 kV.

### Antifungal susceptibility testing by broth microdilution

Broth microdilution was performed in 96-well plates following Clinical and Laboratory Standards Institute guidelines for yeasts (M27-A3) and filamentous fungi (M38-A2). Fungal inocula were prepared from colonies to a final concentration of 0.5 × 10^3^ to 2.5 × 10^3^ CFU/mL for yeasts and 0.4 × 10^4^ to 5 × 10^4^ CFU/mL for molds. All tests used RPMI-1640 (Sigma-Aldrich) supplemented with 0.165 M 3-(N-morpholino)propoanesulfonic acid (MOPS) and 2% D-glucose, except for *C. neoformans*, where YNB (Sigma-Aldrich) supplemented with 0.165 M MOPS and 0.5% D-glucose was used. Assays in serum used media supplemented with either 10% human or fetal bovine serum (FBS, Sigma-Aldrich). Higher serum concentrations (50% and 90%) were tested but found to inhibit fungal growth too extensively for reliable minimum inhibitory concentration (MIC) determination. Plates were incubated at 35°C for 24 h (*S. cerevisiae* and *C. albicans*) or 48 h (*C. neoformans*, *A. fumigatus*, and *M. circinelloides*). The MIC was determined as the lowest drug concentration that inhibited 100% growth and was read visually or by absorbance at 600 nm (BioTek Elx800) for assays with human serum. Assays were performed at least twice, and reported values are the mode. For time-kill curves, CFU per milliliter was measured at 0, 3, 6, and 12 h by backplating on agar plates.

### Drug interaction testing and modeling

Diagonal-sampling checkerboard assays were used to determine pairwise interactions between AMB and test proteins (27). Full checkerboard assays were subsequently performed on AMB in combination with DewY, EAS_Δ15_, and Ub against *S. cerevisiae*. Serial twofold dilutions of AMB and each interacting protein were prepared in 96-well plates in the horizontal and vertical directions, respectively. Inoculum preparation, media, and incubation conditions followed the conditions for antifungal susceptibility testing outlined above. Results were assessed visually and by absorbance at 600 nm (BioTek Elx800). Fractional inhibitory concentration index (FICI) was calculated based on the Loewe additivity model as fractional inhibitory concentration (FIC) = (MIC_*X*_ / MIC_*Y*_), where MIC_*X*_ is the MIC of the drug alone and MIC_*Y*_ is the MIC of the drug in combination. FICI is the sum of the FIC for each drug. Interactions were classified as synergistic (≤0.5), additive or indifferent (>0.5–4.0), or antagonistic (>4.0).

### Assessment of microscopic morphology following treatment

*C. albicans*, *C. neoformans*, *A. fumigatus*, and *M. circinelloides* cultures were untreated or were treated with 1/2 or 1/4 MIC or FIC combinations of AMB, DewY, Ub, AMB + DewY or AMB + Ub. Inoculum preparation, media, and incubation conditions followed the conditions for antifungal susceptibility testing outlined above. At 24 h (*C. albicans*) or 48 h (*C. neoformans*, *A. fumigatus*, and *M. circinelloides*), test wells were examined and photographed using an IS10000 Inverted Microscope (Luminoptic) at 40× using ISCapture Imaging software (Tucsen Photonics).

### Calculation and visualization of exposed surface patches on the proteins

Surface hydrophobic maps of the proteins DewY (PDB ID: 2LSH), EAS_Δ15_ (PDB ID: 2FMC), Ub (PDB ID: 1UBQ), α-lac (PDB ID: 1F6S), and BSA (PDB ID: 3V03) were generated, and the percentage of exposed hydrophobic patches on the surface of the proteins was calculated using Swiss PDB Viewer (https://spdbv.unil.ch/) (25, 28). Image showing the surface hydrophobicity for DewY was generated using ChimeraX with the surface displayed according to molecular hydrophobicity potential.

## RESULTS

### Hydrophobins and ubiquitin increase the solubility of AMB in solution

**Fig 1:**
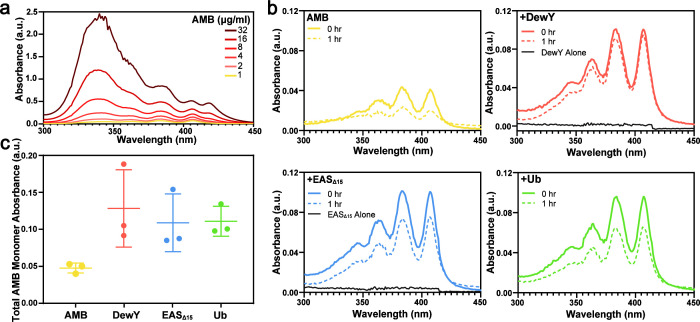
Stabilization of AMB in the presence of the proteins DewY, EAS_Δ15_, and Ub. (**a**) Absorbance spectrum of AMB in water at increasing concentrations (1–32 μg/mL) shows three monomer peaks (365, 385, and 409 nm) and an aggregate peak (348 nm). (**b**) Representative absorbance spectrum of AMB (1 µg/mL) in the presence or absence of DewY, EAS_Δ15_, or Ub before and after 1 h incubation at room temperature. (**c**) Initial total absorbance of AMB monomers (*A*_365_ + *A*_385_ + *A*_409_) in the absence (AMB) and presence of DewY, EAS_Δ15_, and Ub shown as mean ± standard deviation (*n* = 3).

The distinctive absorbance peaks of AMB were utilized to monitor its solubility and self-association: peaks at 409, 385, and 365 nm reflect the presence of monomeric AMB, while a peak at 348 nm is characteristic of AMB aggregates (29, 30). We employed UV-vis spectroscopy and monitored the ratio between the monomer peak at 409 nm and the aggregate peak at 348 nm (*A*_348/409_) to determine AMB solubility and aggregation in water across increasing drug concentrations (1–32 µg/mL). At all concentrations tested, absorbance spectra consistently displayed the monomer and aggregate peaks (Fig. 1a). As expected, the amplitude of all peaks increased with higher AMB concentrations, with a relatively greater increase in the aggregate peak at higher concentrations. An aggregation ratio (*A*_348/409_) of <1 is characteristic of the monomeric form of AMB (31). The aggregate ratios increased from 0.76 at 1 µg/mL to 4.17 at 32 µg/mL, indicating a transition from monomers to aggregates at higher concentrations.

We assessed the ability of the hydrophobin proteins DewY and EAS_Δ15_ to stabilize AMB in the monomeric state. His_6_-Ub was included as a control protein. UV-vis measurements were collected for 1 h from 1 µg/mL AMB solutions prepared with or without protein (Fig. 1b and c). At this concentration and in the absence of any protein, AMB was monomeric but poorly soluble, as indicated by comparatively low peak absorbance values, which decreased further over time due to the formation of insoluble aggregates. In the presence of both hydrophobin proteins, AMB remained soluble and stabilized in its monomeric form. DewY was more effective in stabilizing the monomeric form in solution, as indicated by a smaller decline in absorbance over time. Unexpectedly, the non-hydrophobin protein Ub also exhibited a similar monomer stabilizing and solubilizing effect, indicating that stabilization of the monomeric form of aggregation-prone AMB in aqueous solutions is not unique to hydrophobin proteins.

### Hydrophobins and ubiquitin enhance the efficacy of AMB

**Fig 2:**
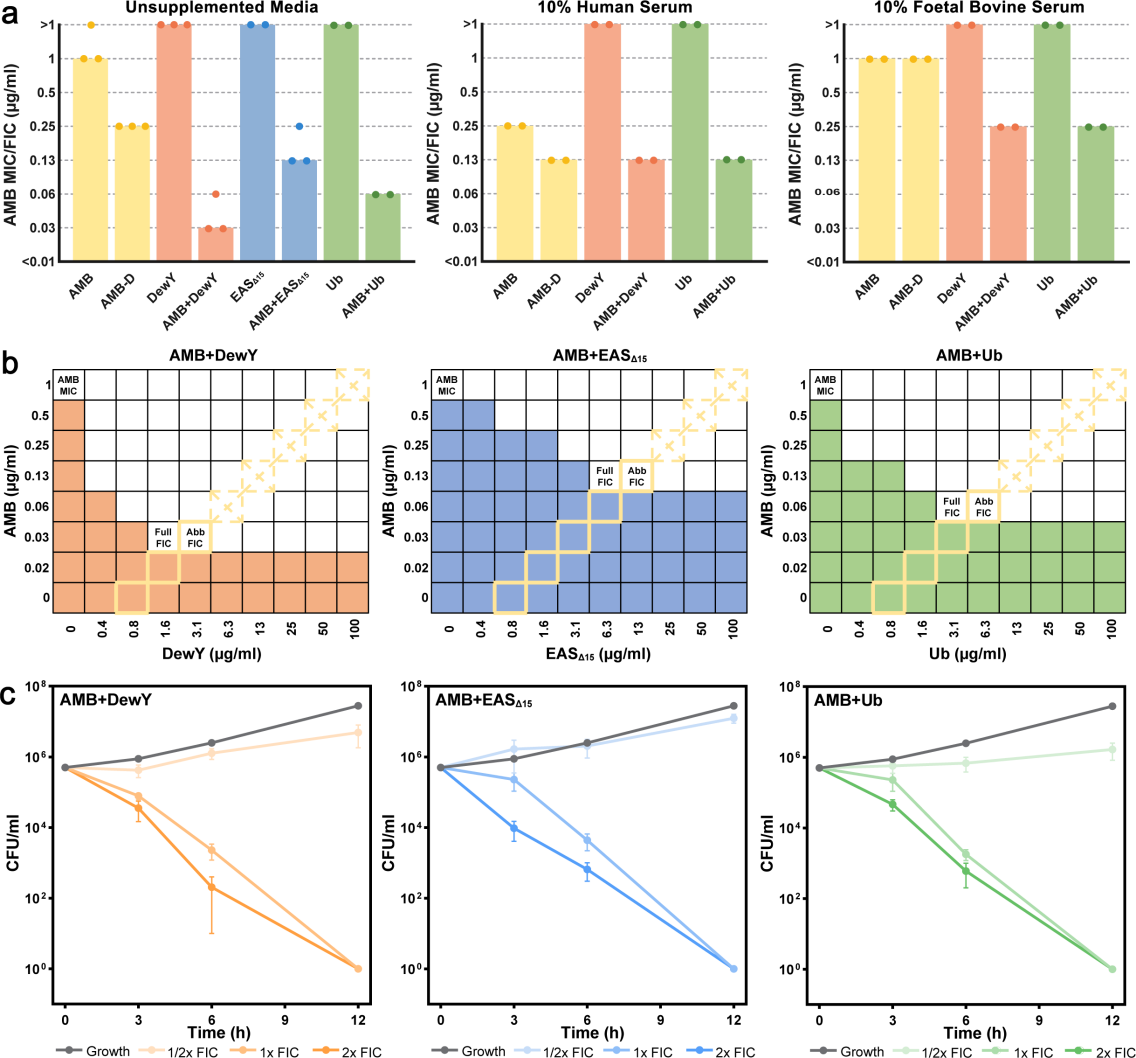
Synergistic interaction of AMB with proteins in *Saccharomyces cerevisiae*. (**a**) The concentration of AMB required to inhibit growth when used alone or in the presence of DewY, EAS_Δ15_, or Ub determined using a diagonal-sampling checkerboard method (24). Data are shown for unsupplemented media (left) or media supplemented with 10% human (middle) or fetal bovine (right) serum. Minimum inhibitory concentration (MIC, the concentration of drug required to inhibit growth) is shown for AMB or AMB-D alone, while fractional inhibitory concentration (FIC, the concentration of drug required to inhibit growth in combination with another agent) is shown for AMB + protein pairs. Bars represent the mode of at least two biological replicates. (**b**) Full checkerboard assays performed to assess the interaction between AMB and DewY, EAS_Δ15_, or Ub. Colored cells denote growth, and white cells denote 100% inhibition. MIC values for each agent and FIC values for the combination of agents (full FIC) are indicated. DewY, EAS_Δ15_, and Ub had no achievable MIC. Concentrations previously tested using the abbreviated checkerboard method are denoted with yellow borders, with areas of growth denoted as dashed crosses, and the abbreviated FIC values (abb FIC) are indicated. (**c**) Time-kill assays of AMB in combination with DewY, EAS_Δ15_, and Ub over 12 h using the 1/2×, 1×, and 2× FIC determined via full checkerboard assays (*n* = 3). Error bars show the SEM.

To determine whether the increased stabilization of the monomeric form of AMB in the presence of a hydrophobin protein or ubiquitin translated to a reduction in the inhibitory dose required for fungal cells, AMB-protein pairs were tested against *Saccharomyces cerevisiae* using a diagonal-sampling checkerboard assay with a starting concentration of 1 µg/mL AMB and 100 µg/mL protein. The resulting FICs are shown in Fig. 2a. The MIC of AMB alone was 1 µg/mL, and as expected, neither the hydrophobin proteins DewY and EAS_Δ15_ nor the control protein Ub alone affected fungal growth (MIC >50 µg/mL). AMB-D, which has a higher solubility in aqueous solution, had an MIC of 0.25 µg/mL, fourfold lower than AMB. All three AMB-protein pairs were found to be remarkably effective in combination, with AMB + DewY achieving a 32-fold reduction in the required concentration of AMB (FIC = 0.03125), AMB + EAS_Δ15_ achieving an 8-fold reduction (FIC = 0.125) and AMB + Ub achieving a 16-fold reduction (FIC = 0.0625).

To test the efficacy of these combinations in serum, which is a complex mixture containing numerous proteins, experiments were repeated with supplementation of either 10% human serum or FBS. In human serum, the MIC of AMB was reduced fourfold to 0.25 µg/mL, and AMB-D, AMB + DewY, and AMB + Ub achieved another twofold reduction. However, human serum alone reduced fungal growth by approximately 60% relative to controls, suggesting that these results may be confounded by other variables that may alter the performance of AMB-protein pairs. Unlike 10% human serum, 10% FBS did not inhibit fungal growth, and the MICs of AMB and AMB-D were both 1 µg/mL. Under these conditions, AMB + DewY and AMB + Ub achieved a fourfold reduction in AMB (FIC = 0.25), indicating continued although somewhat reduced efficacy in the presence of other FBS components. Attempts to increase serum concentration to 50% and 90% resulted in near-complete fungal growth inhibition, making accurate MIC determination unfeasible.

Full checkerboard assays were next performed to explore the interaction profile of AMB-protein pairs further (Fig. 2b). These assays revealed that the same fold reduction in AMB concentration could be achieved with half the amount of each starting protein (“full FIC”) compared to the tests using the abbreviated (abb) checkerboard assays (“abb FIC”). Time-kill assays performed using the updated FIC values (Fig. 2c) showed that AMB-protein pairs at 1× and 2× FIC resulted in 100% cell death within 12 h. At 1/2× FIC, fungal proliferation was delayed relative to the growth control.

### Protein-mediated potentiation is unique to AMB and is not observed with other antifungal drugs

To determine if these three proteins interact with other antifungals, DewY, EAS_Δ15_, and Ub were tested with the polyene NYS, the azole FLC, and the echinocandin CAS, in addition to AMB-D for comparison. The same diagonal-sampling checkerboard method was used with a starting concentration of 1 µg/mL AMB and 50 µg/mL protein. The FICIs for each combination, individual FICs, and AMB fold decrease are listed in Table 1. Interestingly, AMB-D was still potentiated by the three proteins but to a lesser degree (FICI = 0.125–0.5) compared to AMB (FICI = 0.0625–0.25). For NYS + DewY, the FICI was 1, indicating a twofold reduction in the NYS concentration required to achieve inhibition. Although a much smaller decrease than seen with AMB, this indicates that another drug in the same polyene drug class displays some interaction with this protein. All other NYS combinations and all FLC and CAS combinations achieved a no-fold reduction (FICI = 2), indicating no interaction.

**TABLE 1:** Effect of commonly used antifungal drugs in combination with hydrophobins DewY and EAS_Δ15_ and human ubiquitin against *Saccharomyces cerevisiae*

Protein	Drug FIC ^*a*^ (µg/mL)	AMB fold decrease	Protein FIC (µg/mL)	FICI ^*b*^	Synergy ^*c*^
Amphotericin B					
AMB alone	1	–	–	–	–
+DewY	0.03125	32	1.5625	0.0625	Synergistic
+EAS_Δ15_	0.125	8	6.25	0.25	Synergistic
+Ubiquitin	0.0625	16	3.125	0.125	Synergistic
Amphotericin B (deoxycholate)^*d*^					
AMB-D alone	0.25	–	–	–	–
+DewY	0.015625	16	1.5625	0.125	Synergistic
+EAS_Δ15_	0.0625	4	6.25	0.5	Synergistic
+Ubiquitin	0.03125	8	3.125	0.25	Synergistic
Nystatin					
NYS alone	8	–	–	–	–
+DewY	4	2	6.25	1	Indifferent
+EAS_Δ15_	8	1	12.5	2	Indifferent
+Ubiquitin	8	1	12.5	2	Indifferent
Fluconazole					
FLC alone	8	–	–	–	–
+DewY	8	1	25	2	Indifferent
+EAS_Δ15_	8	1	25	2	Indifferent
+Ubiquitin	8	1	25	2	Indifferent
Caspofungin					
CAS alone	16	–	–	–	–
+DewY	16	1	50	2	Indifferent
+EAS_Δ15_	16	1	50	2	Indifferent
+Ubiquitin	16	1	50	2	Indifferent

^*a*^ FIC is shown for combinations; MIC is shown for antifungal drugs alone.

^*b*^ As proteins had no achievable MIC, the highest concentration tested (50 µg/mL) was used for the calculation of FICI.

^*c*^ ≤0.5 = synergistic; >0.5–4.0 = indifferent; >4 = antagonistic.

^*d*^ Dashes where AMB was tested alone indicate no collection of fold-change data.

### Monomer and rodlet forms of DewY interact with AMB to produce similar results

**Fig 3:**
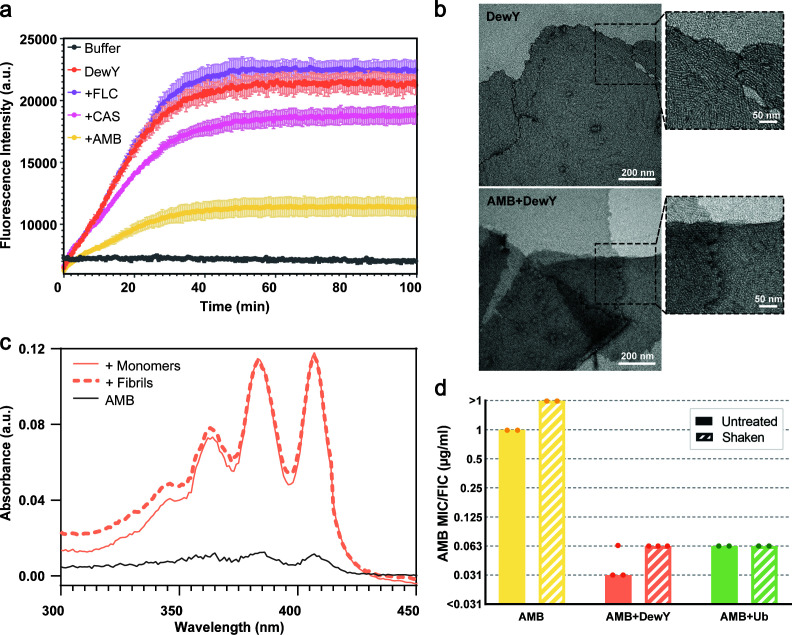
Interaction of AMB with DewY rodlets. (**a**) Rodlet assembly of DewY (50 µg/mL) with shaking was monitored via ThT fluorescence in the presence or absence of FLC, CAS, or AMB. Three replicates of each sample are presented as mean ± standard deviation. (**b**) TEM images of ThT assay samples containing DewY in the presence and absence of AMB. Higher magnification images reveal the packing of rodlets to form monolayers. (**c**) Absorbance spectrum of AMB (1 µg/mL) in the presence of DewY monomer or rodlets. AMB binds to hydrophobin rodlets. (**d**) The concentration of AMB required to inhibit the growth of *Saccharomyces cerevisiae* alone or in the presence of DewY and Ub (100 µg/mL). Solid bars indicate untreated samples, and striped bars indicate shaken samples; all bars represent the mode of at least two biological replicates.

The DewY hydrophobin protein, which showed the greatest ability to stabilize monomeric AMB and achieved the largest fold reduction in AMB against fungal cells, was chosen for further detailed examination of the interaction with AMB. Class I hydrophobins such as DewY and EAS_Δ15_ are known to self-assemble and form amyloid fibril structures known as rodlets, and these further assemble into amphipathic monolayers that facilitate interactions between fungal structures (e.g., spores) and environmental or host surfaces (15, 18, 32). The rodlet assembly of hydrophobins can be monitored using the fluorescent dye ThT, which fluoresces upon binding to the cross-β structure characteristic of amyloid fibrils (18, 33). To assess the interaction of AMB with DewY during rodlet assembly, ThT fluorescence was measured in the presence and absence of AMB (Fig. 3a). When solutions of DewY were agitated, a marked increase in ThT fluorescence was observed, consistent with rodlet assembly. The addition of AMB reduced both the rate of increase and the final intensity of ThT fluorescence. The presence of CAS had a smaller effect, and FLC did not alter the ThT fluorescence profile. Transmission electron microscopy demonstrated that DewY rodlet formation occurred in both the presence and absence of AMB (Fig. 3b), suggesting that AMB prevents ThT from binding to the rodlet surface but does not inhibit DewY rodlet assembly. Similar effects have been observed with other low-molecular-weight molecules tested as inhibitors of amyloid assembly, where ThT binding is disrupted by competing compounds (34).

The effect of the different monomeric and rodlet forms of DewY on the stability of AMB was further compared by UV-vis spectroscopy and antifungal susceptibility testing. The absorbance spectra of AMB in the presence of DewY in monomeric and rodlet forms showed comparable high AMB absorbance peaks, indicating a similar stabilization capacity (Fig. 3c). Antifungal susceptibility testing against *S. cerevisiae* showed that DewY in rodlet form (FIC = 0.625) was almost as effective as DewY in monomer form (FIC = 0.03125), achieving a 16-fold reduction in AMB (Fig. 3d). These results suggest that AMB binds similarly to DewY monomer and rodlets without impacting the self-assembly of the protein, and both stabilize the drug in its monomeric form.

### Stabilization of AMB can be achieved with a diverse range of proteins and persists for up to 48 h

**Fig 4:**
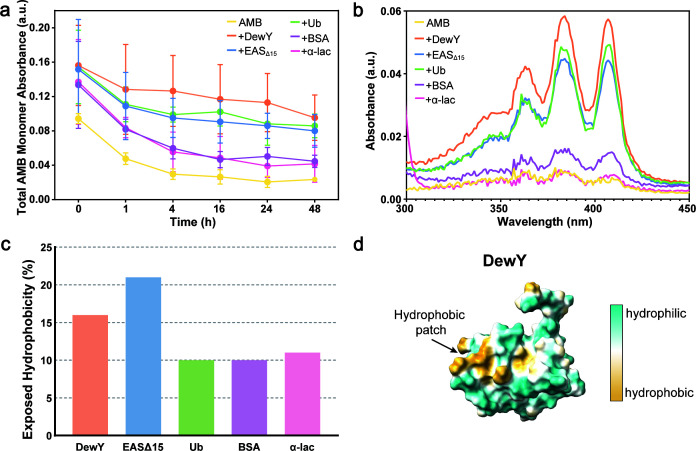
Stabilization of AMB over 48 h in the presence of proteins. (**a**) Total absorbance of AMB monomers (*A*_409_ + *A*_385_ + *A*_365_) in the absence and presence of the proteins (50 µg/mL) DewY (orange), EAS_Δ15_ (blue), Ub (green), BSA (purple) or α-lac (pink) over a 48 h incubation in water. Each point is shown as mean ± standard deviation (*n* = 3). (**b**) Representative absorbance spectrum of AMB (1 µg/mL) in the absence or presence of the proteins after 48 h. (**c**) Extent of exposed surface area that is hydrophobic for each protein calculated using Swiss PDB viewer. (**d**) Surface representation of DewY (PDB ID:2LSH) visualized in ChimeraX, with the surface displayed as molecular hydrophobicity potential with hydrophilic (blue) and hydrophobic (yellow) regions. Example hydrophobic patch indicated with an arrow.

We next tested additional proteins for the ability to stabilize AMB. BSA and α-lac were evaluated alongside DewY, EAS_Δ15_, and Ub. AMB monomer was monitored over 48 h by measuring the combined absorbance of monomer peaks (*A*_409_ + *A*_385_ + *A*_365_) in the UV-vis spectra. All protein-AMB combinations initially showed higher monomer absorbance than AMB alone, but only DewY, EAS_Δ15_, and Ub showed sustained higher absorbance than AMB alone throughout the incubation period in all samples (Fig. 4a and b). The inclusion of DewY resulted in a total monomer absorbance of 0.156 at 0 h (compared to 0.095 for AMB alone) and 0.092 at 48 h (compared to 0.023 for AMB), demonstrating a significant and prolonged stabilization effect. The physicochemical basis for the stabilization of the AMB monomer by the proteins was explored by calculating and visualizing the total area of exposed hydrophobicity on their surfaces. Exposed hydrophobic patches make up 10% of the surfaces of BSA and Ub, 11% for α-lac, 16% for DewY, and 21% for EAS_Δ15_ (Fig. 4c). No clear relationship was observed between exposed hydrophobicity and the ability of each protein to stabilize the AMB monomer; however, DewY does exhibit a clear contiguous hydrophobic patch which may be the site of interaction with AMB (Fig. 4d).

Antifungal efficacy was assessed against four clinically relevant fungal pathogens: *Cryptococcus neoformans*, *Candida albicans*, *Aspergillus fumigatus*, and *Mucor circinelloides*. FICIs, individual FICs, and AMB fold-reduction values were determined using a diagonal-sampling checkerboard method with a starting concentration of 1 µg/mL AMB and 50 µg/mL protein (Table 2). All AMB-protein pairs showed improved efficacy compared to AMB alone against all species tested (FICI = 0.0625–0.5). Among the tested combinations, AMB + DewY was consistently the most effective (FICI = 0.0625–0.125), achieving a 32-fold reduction in AMB against *C. neoformans*, *C. albicans*, and *M. circinelloides* and 16-fold reduction against *A. fumigatus*. Conversely, AMB + EAS_Δ15_ was the least effective (FICI = 0.125–0.5), with a 16-fold reduction in AMB against *C. neoformans* and *C. albicans*, an 8-fold reduction against *M. circinelloides*, and a 4-fold reduction against *A. fumigatus*. Ub, α-lac, and BSA displayed intermediate efficacy, with consistent ranking across all species, indicating that the drug-protein interaction primarily drives efficacy rather than species-specific factors.

**TABLE 2:** Effect of AMB-protein pairs on fungal pathogens

Protein	Drug FIC ^*a*^ (µg/mL)	AMB fold decrease	Protein FIC (µg/mL)	FICI ^*b*^	Synergy ^*c*^
*Cryptococcus neoformans*					
AMB alone	1	–	–	–	–
+DewY	0.03125	32	1.5625	0.0625	Synergistic
+EAS_Δ15_	0.0625	16	3.125	0.125	Synergistic
+Ubiquitin	0.03125	32	1.5625	0.0625	Synergistic
+α-Lactalbumin	0.0625	16	3.125	0.125	Synergistic
+Bovine serum albumin	0.03125	32	1.5625	0.0625	Synergistic
*Candida albicans*					
AMB alone	1	–	–	–	–
+DewY	0.03125	32	1.5625	0.0625	Synergistic
+EAS_Δ15_	0.0625	16	3.125	0.125	Synergistic
+Ubiquitin	0.0625	16	3.125	0.125	Synergistic
+α-Lactalbumin	0.0625	16	3.125	0.125	Synergistic
+Bovine serum albumin	0.0625	16	3.125	0.125	Synergistic
*Aspergillus fumigatus*					
AMB alone	1	–	–	–	–
+DewY	0.0625	16	3.125	0.125	Synergistic
+EAS_Δ15_	0.25	4	12.5	0.5	Synergistic
+Ubiquitin	0.125	8	6.25	0.25	Synergistic
+α-Lactalbumin	0.125	8	6.25	0.25	Synergistic
+Bovine serum albumin	0.0625	16	3.125	0.125	Synergistic
*Mucor circinelloides*					
AMB alone	1	–	–	–	–
+DewY	0.03125	32	1.5625	0.0625	Synergistic
+EAS_Δ15_	0.125	8	6.25	0.25	Synergistic
+Ubiquitin	0.03125	32	1.5625	0.0625	Synergistic
+α-Lactalbumin	0.0625	16	3.125	0.125	Synergistic
+Bovine serum albumin	0.03125	32	1.5625	0.0625	Synergistic

^*a*^ FIC is shown for combinations; MIC is shown for AMB alone.

^*b*^ As proteins had no achievable MIC, the highest concentration tested (50 µg/mL) was used for the calculation of FICI.

^*c*^ ≤0.5 = synergistic; >0.5–4.0 = indifferent; >4 = antagonistic.

### AMB-protein induces the same morphological damage to cells and hyphae as AMB alone

**Fig 5:**
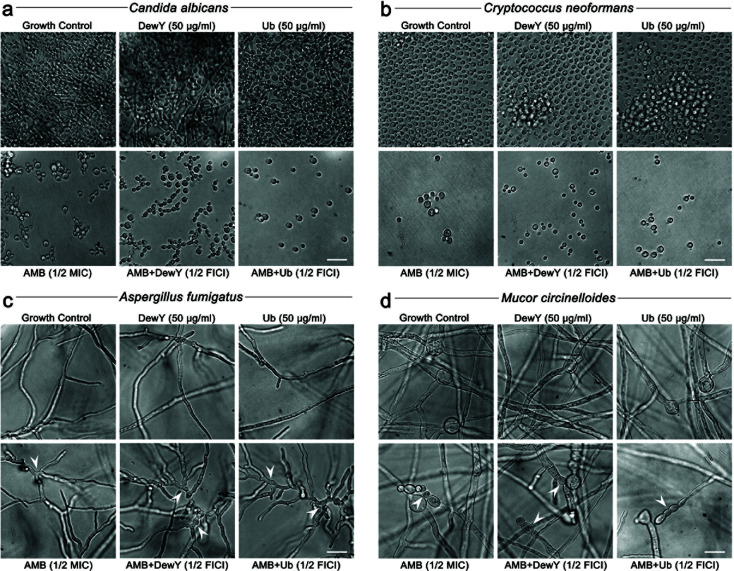
Morphological changes induced in fungal pathogens by sub-inhibitory concentrations of AMB and AMB-protein pairs. Light microscopy images of (**a**) *Candida albicans*, (**b**) *Cryptococcus neoformans*, (**c**) *Aspergillus fumigatus*, and (**d**) *Mucor circinelloides* treated with 1/2 MIC of AMB (0.5 µg/mL), 50 µg/mL of DewY or Ub, or 1/2 FIC of AMB + DewY (0.016–0.031 µg/mL AMB) or AMB + Ub (0.016–0.063 µg/mL AMB) after 48 h compared to an untreated growth control. Scale bars = 10 µM. White arrowheads indicate morphological changes following treatment.

Microscopic examination revealed that AMB-protein combinations induced similar morphological changes in fungal cells as AMB alone but at lower drug concentrations (Fig. 5). A treatment concentration of 1/2 MIC or 1/2 FIC was chosen to allow the growth and reproduction of cells while still subjecting them to stress. In *Candida albicans*, AMB and AMB-protein treatments inhibited hyphal production, with AMB + DewY yielding a mix of elongated opaque and round white cells, while AMB + Ub primarily produced the more virulent round white cells (Fig. 5a). In *Cryptococcus neoformans*, AMB-protein treatments reduced the proportion of enlarged cells compared to AMB alone (Fig. 5b). In *Aspergillus fumigatus*, AMB and AMB-protein treatments caused irregularly twisted and highly branched hyphae, with stronger effects seen in AMB + DewY and AMB + Ub (Fig. 5c). Similarly, in *Mucor circinelloides*, AMB-protein treatments reduced sporangia formation, with the remaining sporangia often deformed or swollen (Fig. 5d). Overall, AMB-protein treatments induced the same types of morphological changes as AMB alone but at reduced drug concentrations, indicating that equivalent damage can be achieved using less AMB when it is formulated with proteins.

## DISCUSSION

Since its discovery in the 1950s, AMB has been a cornerstone broad-spectrum antifungal agent. Its primary mechanism involves disrupting and puncturing ergosterol-containing fungal membranes (1), but it can also interact with cholesterol in host cells, which contributes to significant toxicity (30). Additionally, the poor solubility of AMB and tendency to form aggregates present challenges to its pharmacokinetics, where multimolecular aggregates complicate intravenous delivery (29). This study investigated the potential of fungal hydrophobins and other proteins to stabilize AMB in its monomeric state, enhancing its solubility and antifungal efficacy. Hydrophobins, with their relatively large and exposed hydrophobic surfaces, were hypothesized to be uniquely suited to encapsulate or stabilize AMB in aqueous environments (15). However, while hydrophobins were very effective, similar effects were observed with other non-hydrophobin proteins. These findings align with previous studies that reported the potentiation of AMB by individual proteins, such as the milk protein lactoferrin and BSA (9, 35). Our study expands this understanding by demonstrating that protein-mediated stabilization of AMB is not limited to specific proteins but could represent a broader strategy applicable across a range of proteins.

**Fig 6:**
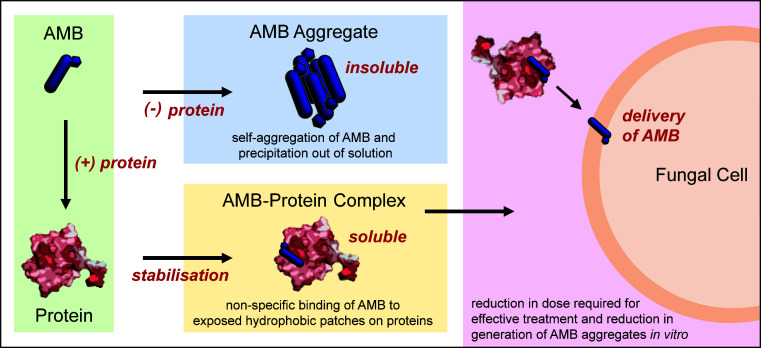
Schematic illustrating the proposed mechanism of AMB-protein synergy. AMB binds non-specifically to exposed hydrophobic patches on the surface of proteins, stabilizing it in monomeric form in solution.

Our results suggest a non-specific stabilization mechanism that involves transient interactions between AMB monomers and exposed hydrophobic patches on proteins, thereby reducing the rate and extent of aggregation, as summarized in Fig. 6. This is supported by the ThT assays, where ThT bound to amyloid rodlets was displaced by AMB. The extent of hydrophobicity of the proteins alone does not predict efficacy, as despite its larger hydrophobic surface (Fig. 4d), the AMB-EAS_Δ15_ combination was not as effective as the AMB-DewY and AMB-BSA pairings. Structural factors like binding groove accessibility may play a role; for example, the BSA surface is known to display several hydrophobic grooves that bind fatty acids, and this may explain its superior performance (36). Of note, protein binding did not alter the underlying mechanism of action of AMB. Microscopy revealed consistent damage by AMB alone and AMB-protein combinations, and there was a consistent ranking in the order of efficacy of AMB-protein pairs against various fungal pathogens. Together, these findings suggest that the individual nature of the protein used to stabilize AMB determines the level of potentiation, which may be influenced by both hydrophobic and structural features.

In the body, AMB binds to various plasma proteins such as lipoproteins and albumin, influencing its distribution, delivery to host tissues, and toxicity (37–39). Consistent with the results presented here, serum albumin has been reported to interact with both AMB and NYS and polyene drugs, increasing the critical aggregation concentration of both and lowering hemolytic activity (39, 40). As well as reducing aggregation, the non-specific binding of AMB to these proteins *in vivo* is thought to reduce the proportion of free unbound AMB present in the body. A study comparing deoxycholate and liposomal formulations of AMB found that liposomal AMB exhibited a greater degree of protein binding, which aligns with a lowered distribution to the kidneys and reduced nephrotoxicity of liposomal AMB and suggests that the free unbound drug drives these processes (41).

Pre-binding of AMB to specific proteins with desirable properties in pharmaceutical formulations could present a promising approach to replicate the beneficial effects of *in vivo* protein interactions, in addition to reducing the required dose by monomer stabilization. Such pre-binding could enhance the effective solubility of AMB in the bloodstream, aid systemic transport, and reduce the levels of free diffusible drug, thereby mitigating off-target effects associated with aggregation. Potential negative effects may include altered pharmacokinetics or drug clearance, possibly impacting efficacy or duration of action (42). For example, reduced AMB mobility might hinder its ability to penetrate certain tissues or cross biological barriers to reach infection sites, or even concentrate the drug at certain sites leading to increased localized toxicity. If these adverse effects could be controlled by selectively pre-binding AMB to proteins with desirable characteristics, toxic effects could be mitigated and targeted delivery could be enhanced.

### Conclusion

This study provides new insights into the role of protein interactions in modulating AMB solubility and stabilizing it in its less toxic monomeric state. As a critical yet toxic antifungal, these findings offer a potential avenue for enhancing the clinical effectiveness of AMB by decreasing its therapeutic dose and reducing the formulation of damaging aggregates. While our study focuses on *in vitro* characterization, further research is needed to assess the safety and efficacy of particular AMB-protein complexes in host cells and animal models. Investigating their stability and functionality under physiological conditions, their pharmacokinetics, and their ability to reach infection sites effectively will be essential for translation into clinical applications. Exploring protein-engineering approaches to optimize the AMB-protein interactions could further improve drug delivery and reduce off-target effects. These findings may also inform broader applications for protein-drug interactions in other therapeutic areas where drug solubility and toxicity are major limitations.

## Supplementary Material

Reviewer comments

## Data Availability

All data generated or analyzed during this study are included in this published article.

## SUPPLEMENTAL MATERIAL

OPEN PEER REVIEW reviewer-comments.pdf An accounting of the reviewer comments and feedback.

ASM does not own the copyrights to Supplemental Material that may be linked to, or accessed through, an article. The authors have granted ASM a non-exclusive, world-wide license to publish the Supplemental Material files. Please contact the corresponding author directly for reuse.

## References

[1] Gray KC, Palacios DS, Dailey I, Endo MM, Uno BE, Wilcock BC, Burke MD. 2012. Amphotericin primarily kills yeast by simply binding ergosterol. Proc Natl Acad Sci USA 109:2234–2239. doi:10.1073/pnas.1117280109 22308411 PMC3289339

[2] Sawaya BP, Briggs JP, Schnermann J, Sawaya BP, Briggs JP, Schnermann J. 1995. Amphotericin B nephrotoxicity: the adverse consequences of altered membrane properties. J Am Soc Nephrol 6:154–164. doi:10.1681/ASN.V62154 7579079

[3] Hamill RJ. 2013. Amphotericin B formulations: a comparative review of efficacy and toxicity. Drugs 73:919–934. doi:10.1007/s40265-013-0069-4 23729001

[4] Stone NRH, Bicanic T, Salim R, Hope W. 2016. Liposomal Amphotericin B (AmBisome(®)): a review of the pharmacokinetics, pharmacodynamics, clinical experience and future directions. Drugs 76:485–500. doi:10.1007/s40265-016-0538-7 26818726 PMC4856207

[5] Maji A, Soutar CP, Zhang J, Lewandowska A, Uno BE, Yan S, Shelke Y, Murhade G, Nimerovsky E, Borcik CG. 2023. Tuning sterol extraction kinetics yields a renal-sparing polyene antifungal. Nature New Biol 623:1079–1085. doi:10.1038/s41586-023-06710-4 PMC10883201 37938782

[6] Seidel D, Wurster S, Jenks JD, Sati H, Gangneux J-P, Egger M, Alastruey-Izquierdo A, Ford NP, Chowdhary A, Sprute R, Cornely O, Thompson GR 3rd, Hoenigl M, Kontoyiannis DP. 2024. Impact of climate change and natural disasters on fungal infections. Lancet Microbe 5:e594–e605. doi:10.1016/S2666-5247(24)00039-9 38518791

[7] Gebremariam T, Gu Y, Singh S, Kitt TM, Ibrahim AS. 2021. Combination treatment of liposomal Amphotericin B and isavuconazole is synergistic in treating experimental mucormycosis. J Antimicrob Chemother 76:2636–2639. doi:10.1093/jac/dkab233 34263306 PMC8446914

[8] Pereira TC, do Carmo PHF, de Menezes RT, de Oliveira HC, de Oliveira LD, Junqueira JC, Scorzoni L. 2023. Synergistic effect of the verapamil and Amphotericin B against Cryptococcus neoformans. Folia Microbiol 68:999–1004. doi:10.1007/s12223-023-01104-5 37950840

[9] Fernandes KE, Weeks K, Carter DA. 2020. Lactoferrin is broadly active against yeasts and highly synergistic with Amphotericin B. Antimicrob Agents Chemother 64:e02284-19. doi:10.1128/AAC.02284-19 32094132 PMC7179636

[10] Fernandes KE, Payne RJ, Carter DA. 2020. Lactoferrin-derived peptide lactofungin is potently synergistic with Amphotericin B. Antimicrob Agents Chemother 64:e00842-20. doi:10.1128/AAC.00842-20 32690642 PMC7508585

[11] Janout V, Schell WA, Thévenin D, Yu Y, Perfect JR, Regen SL. 2015. Taming Amphotericin B. Bioconjug Chem 26:2021–2024. doi:10.1021/acs.bioconjchem.5b00463 26340430 PMC7263395

[12] Legrand P, Romero EA, Cohen BE, Bolard J. 1992. Effects of aggregation and solvent on the toxicity of Amphotericin B to human erythrocytes. Antimicrob Agents Chemother 36:2518–2522. doi:10.1128/AAC.36.11.2518 1489196 PMC284364

[13] Torrado JJ, Espada R, Ballesteros MP, Torrado-Santiago S. 2008. Amphotericin B formulations and drug targeting. J Pharm Sci 97:2405–2425. doi:10.1002/jps.21179 17893903

[14] Yamashita K, Janout V, Bernard EM, Armstrong D, Regen SL. 1995. Micelle/monomer control over the membrane-disrupting properties of an amphiphilic antibiotic. J Am Chem Soc 117:6249–6253. doi:10.1021/ja00128a011

[15] Linder MB, Szilvay GR, Nakari-Setälä T, Penttilä ME. 2005. Hydrophobins: the protein-amphiphiles of filamentous fungi. FEMS Microbiol Rev 29:877–896. doi:10.1016/j.femsre.2005.01.004 16219510

[16] Aimanianda V, Bayry J, Bozza S, Kniemeyer O, Perruccio K, Elluru SR, Clavaud C, Paris S, Brakhage AA, Kaveri SV, Romani L, Latgé J-P. 2009. Surface hydrophobin prevents immune recognition of airborne fungal spores. Nature New Biol 460:1117–1121. doi:10.1038/nature08264 19713928

[17] Bayry J, Aimanianda V, Guijarro JI, Sunde M, Latgé JP. 2012. Hydrophobins--unique fungal proteins. Plos Pathog 8:e1002700. doi:10.1371/journal.ppat.1002700 22693445 PMC3364958

[18] Sunde M, Kwan AHY, Templeton MD, Beever RE, Mackay JP. 2008. Structural analysis of hydrophobins. Micron 39:773–784. doi:10.1016/j.micron.2007.08.003 17875392

[19] Macindoe I, Kwan AH, Ren Q, Morris VK, Yang W, Mackay JP, Sunde M. 2012. Self-assembly of functional, amphipathic amyloid monolayers by the fungal hydrophobin EAS. Proc Natl Acad Sci USA 109:E804–11. doi:10.1073/pnas.1114052109 22308366 PMC3325668

[20] Sunde M, Pham CLL, Kwan AH. 2017. Molecular characteristics and biological functions of surface-active and surfactant proteins. Annu Rev Biochem 86:585–608. doi:10.1146/annurev-biochem-061516-044847 28125290

[21] Song B, Wang W, Jia C, Han Z, Yang J, Yang J, Wu Z, Xu H, Qiao M. 2024. Identification and characterization of a predominant hydrophobin in the edible mushroom grifola frondosa. JoF 10:25. doi:10.3390/jof10010025 PMC10820438 38248935

[22] Haas Jimoh Akanbi M, Post E, Meter-Arkema A, Rink R, Robillard GT, Wang X, Wösten HAB, Scholtmeijer K. 2010. Use of hydrophobins in formulation of water insoluble drugs for oral administration. Colloids Surf B Biointerfaces 75:526–531. doi:10.1016/j.colsurfb.2009.09.030 19836932

[23] Osmanagaoglu FH, Ekmekcioglu A, Ozcan B, Bayram Akcapinar G, Muftuoglu M. 2024. Preparation and characterization of hydrophobin 4-coated liposomes for doxorubicin delivery to cancer cells. Pharmaceuticals (Basel) 17:1422. doi:10.3390/ph17111422 39598333 PMC11597365

[24] Barani M, Mirzaei M, Torkzadeh-Mahani M, Lohrasbi-Nejad A, Nematollahi MH. 2020. A new formulation of hydrophobin-coated niosome as a drug carrier to cancer cells. Materials Science and Engineering: C 113:110975. doi:10.1016/j.msec.2020.110975 32487392

[25] Sarparanta M, Bimbo LM, Rytkönen J, Mäkilä E, Laaksonen TJ, Laaksonen P, Nyman M, Salonen J, Linder MB, Hirvonen J, Santos HA, Airaksinen AJ. 2012. Intravenous delivery of hydrophobin-functionalized porous silicon nanoparticles: stability, plasma protein adsorption and biodistribution. Mol Pharmaceutics 9:654–663. doi:10.1021/mp200611d 22277076

[26] Siddiquee R, Lo V, Johnston CL, Buffier AW, Ball SR, Ciofani JL, Zeng YC, Mahjoub M, Chrzanowski W, Rezvani-Baboli S, Brown L, Pham CLL, Sunde M, Kwan AH. 2023. Surface-induced hydrophobin assemblies with versatile properties and distinct underlying structures. Biomacromolecules 24:4783–4797. doi:10.1021/acs.biomac.3c00542 37747808

[27] Cokol-Cakmak M, Bakan F, Cetiner S, Cokol M. 2018. Diagonal method to measure synergy among any number of drugs. J Vis Exp 136:57713. doi:10.3791/57713 PMC6101960 29985330

[28] Guex N, Peitsch MC. 1997. SWISS-MODEL and the SWISS-PdbViewer: an environment for comparative protein modeling. Electrophoresis 18:2714–2723. doi:10.1002/elps.1150181505 9504803

[29] Kajtár M, Vikmon M, Morlin E, Szejtli J. 1989. Aggregation of Amphotericin B in the presence of gamma-cyclodextrin. Biopolymers 28:1585–1596. doi:10.1002/bip.360280908 2775849

[30] Mazerski J, Grzybowska J, Borowski E. 1990. Influence of net charge on the aggregation and solubility behaviour of Amphotericin B and its derivatives in aqueous media. Eur Biophys J 18:159–164. doi:10.1007/BF02427375 2357943

[31] Gurudevan S, Francis AP, Jayakrishnan A. 2018. Amphotericin B-albumin conjugates: synthesis, toxicity and anti-fungal activity. Eur J Pharm Sci 115:167–174. doi:10.1016/j.ejps.2018.01.017 29325755

[32] Morris VK, Kwan AH, Sunde M. 2013. Analysis of the structure and conformational states of DewA gives insight into the assembly of the fungal hydrophobins. J Mol Biol 425:244–256. doi:10.1016/j.jmb.2012.10.021 23137797

[33] Krebs MRH, Bromley EHC, Donald AM. 2005. The binding of thioflavin-T to amyloid fibrils: localisation and implications. J Struct Biol 149:30–37. doi:10.1016/j.jsb.2004.08.002 15629655

[34] Noormägi A, Primar K, Tõugu V, Palumaa P. 2012. Interference of low-molecular substances with the thioflavin-T fluorescence assay of amyloid fibrils. J Pept Sci 18:59–64. doi:10.1002/psc.1416 22083646

[35] Grela E, Stączek S, Nowak M, Pawlikowska-Pawlega B, Zdybicka-Barabas A, Janik S, Cytryńska M, Grudzinski W, Gruszecki WI, Luchowski R. 2023. Enhanced antifungal activity of Amphotericin B bound to albumin: a “Trojan Horse” effect of the protein. J Phys Chem B 127:3632–3640. doi:10.1021/acs.jpcb.3c01168 37071547 PMC10150355

[36] Ma GJ, Ferhan AR, Jackman JA, Cho NJ. 2020. Conformational flexibility of fatty acid-free bovine serum albumin proteins enables superior antifouling coatings. Commun Mater 1. doi:10.1038/s43246-020-0047-9

[37] Brajtburg J, Elberg S, Bolard J, Kobayashi GS, Levy RA, Ostlund RE Jr, Schlessinger D, Medoff G. 1984. Interaction of plasma proteins and lipoproteins with Amphotericin B. J Infect Dis 149:986–997. doi:10.1093/infdis/149.6.986 6376657

[38] Wasan KM, Morton RE, Rosenblum MG, Lopez-Berestein G. 1994. Decreased toxicity of liposomal Amphotericin B due to association of Amphotericin B with high-density lipoproteins: role of lipid transfer protein. J Pharm Sci 83:1006–1010. doi:10.1002/jps.2600830716 7965656

[39] Hartsel SC, Baas B, Bauer E, Foree LT, Kindt K, Preis H, Scott A, Kwong EH, Ramaswamy M, Wasan KM. 2001. Heat‐induced superaggregation of Amphotericin B modifies its interaction with serum proteins and lipoproteins and stimulation of TNF‐α. J Pharm Sci 90:124–133. doi:10.1002/1520-6017(200102)90:2<124::AID-JPS3>3.3.CO;2-x 11169529

[40] Aramwit P, Yu BG, Lavasanifar A, Samuel J, Kwon GS. 2000. The effect of serum albumin on the aggregation state and toxicity of Amphotericin B. J Pharm Sci 89:1589–1593. doi:10.1002/1520-6017(200012)89:12<1589::aid-jps10>3.0.co;2-6 11042607

[41] Bekersky I, Fielding RM, Dressler DE, Lee JW, Buell DN, Walsh TJ. 2002. Plasma protein binding of amphotericin B and pharmacokinetics of bound versus unbound Amphotericin B after administration of intravenous liposomal Amphotericin B (AmBisome) and Amphotericin B deoxycholate. Antimicrob Agents Chemother 46:834–840. doi:10.1128/AAC.46.3.834-840.2002 11850269 PMC127463

[42] Lindup WE, Orme MC. 1981. Clinical pharmacology: plasma protein binding of drugs. Br Med J (Clin Res Ed) 282:212–214. doi:10.1136/bmj.282.6259.212 PMC1503970 6779954

